# Promoting Cold-Start Items in Recommender Systems

**DOI:** 10.1371/journal.pone.0113457

**Published:** 2014-12-05

**Authors:** Jin-Hu Liu, Tao Zhou, Zi-Ke Zhang, Zimo Yang, Chuang Liu, Wei-Min Li

**Affiliations:** 1 Web Sciences Center, University of Electronic Science and Technology of China, Chengdu, People's Republic of China; 2 Big Data Research Center, University of Electronic Science and Technology of China, Chengdu, People's Republic of China; 3 Alibaba Research Center for Complexity Sciences, Hangzhou Normal University, Hangzhou, People's Republic of China; 4 Alibaba Group, Hangzhou, People's Republic of China; 5 Beijing Baifendian Information Technology Co., Ltd., Beijing, People's Republic of China; Northwestern University, United States of America

## Abstract

As one of the major challenges, cold-start problem plagues nearly all recommender systems. In particular, new items will be overlooked, impeding the development of new products online. Given limited resources, how to utilize the knowledge of recommender systems and design efficient marketing strategy for new items is extremely important. In this paper, we convert this ticklish issue into a clear mathematical problem based on a bipartite network representation. Under the most widely used algorithm in real e-commerce recommender systems, the so-called item-based collaborative filtering, we show that to simply push new items to active users is not a good strategy. Interestingly, experiments on real recommender systems indicate that to connect new items with some less active users will statistically yield better performance, namely, these new items will have more chance to appear in other users' recommendation lists. Further analysis suggests that the disassortative nature of recommender systems contributes to such observation. In a word, getting in-depth understanding on recommender systems could pave the way for the owners to popularize their cold-start products with low costs.

## Introduction

Thanks to the blazing development of Internet, e-commerce has flourished over the past decades. With the online buy-and-sell platforms getting increasingly more available products (e.g., more than a billion products in *taobao.com*), shopping online has become a fashionable style of living and more people choose to purchase on the Internet rather than go to stores. E-commerce makes our life much more convenient, meanwhile, it throws us into a dilemma of information overloads. Facing millions of items online, finding out favourites is rather difficult. As an effective information filtering tool, recommender system is thus of particular significance nowadays [1], [2]. In fact, it has already made considerable contributions to the socioeconomic fields in the past decade. For example, 60% of DVDs rented by *Netflix* are selected based on personalized recommendations, and about a half of sales in *Amazon* are brought by recommendations [2]. Consequently, recommender systems have received huge attentions from both physicists and computer scientists, and many advanced recommendation algorithms are proposed recently, including collaborative filtering [3]–[8], content-based analysis [9]–[11], dimensionality reduction techniques [12]–[14], diffusion-based methods [15]–[22], and so on.

One long-standing challenge, called cold-start problem, has plagued almost all recommender systems. Namely, when new users or items enter the system, there is usually insufficient information to produce reasonable recommendation [23]. Considering this fact, several potential solutions have been raised. The additional content information [23]–[26], tagging information [27]–[29] and cross-domain information [30] can be used to marginally relieve this problem, but they don't work in a purely cold-start setting, where no information is available to form any basis for recommendations. Furthermore, improving diversity and novelty of recommended lists can help new items be pushed out [19], [31], [32].

Practically speaking, as a holder of the recommender system, one can ask for extra information to generate initial profiles on users or items [24], or probe users' preferences by pushing to them some carefully selected items according to complicated algorithms [33]. Both methods are costly and risky. In contrast, an owner would like to popularize his/her new items. An improper method, called “shilling attacks”, injects a number of mendacious users into the system to raise predicted ratings of new items, and thus enhances the possibility of these new items to appear in the recommendation lists [34], [35]. But, it is easily to be detected [36]–[38]. Furthermore, as a wide-spreading market strategy, advertisements are generally preferred and become more and more prosperous [39]. However, to popularize new items costs a lot and imposes an unbearable financial burden for small businesses [40]. As mentioned above, how to promote new items under limited marketing resources is a nontrivial challenge and the knowledge of recommendation algorithm may be helpful. Putting aside operational details, if the marketing activities can bring some purchases of certain users, a smart marketing manager will carefully choose the target users so that these purchases can lead to more exposures in the recommendation lists afterwards.

Taking a stand as a marketing manager, in this paper, we focus on how to promote cold-start items by utilizing the knowledge of recommender systems. The main contributions are threefold: (i) We convert this ticklish problem into a clear mathematical model that ignores some insignificant details. (ii) We show that to push new items to active users, a straightforward strategy that will jump into our mind at the first time, is an unexpectedly poor-performed strategy. (iii) We propose a degree-based solution that outperforms some baseline methods.

## Results

**Figure 1:**
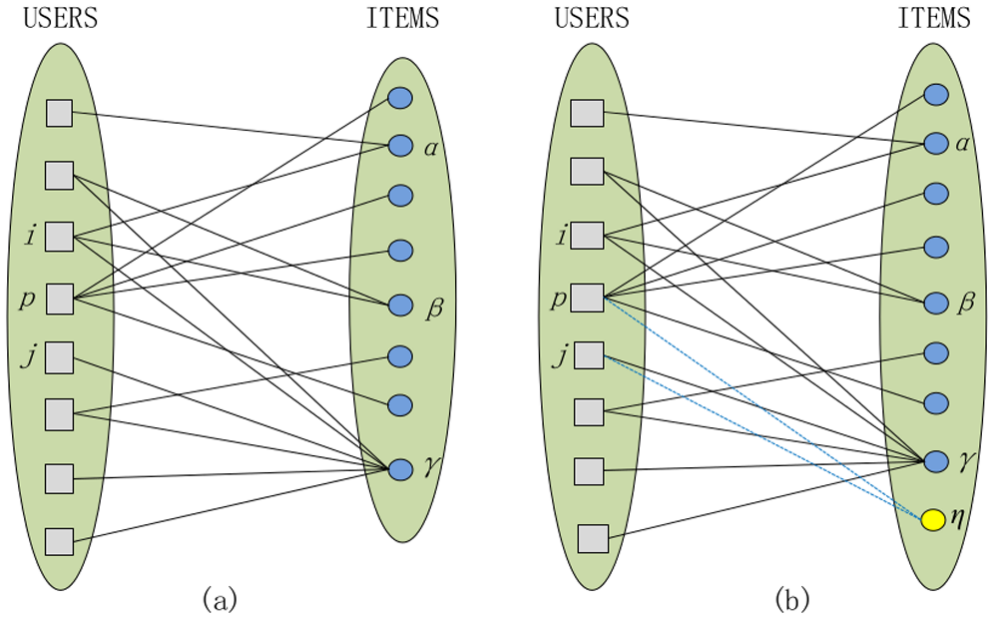
How to add a cold-start item to the user-item bipartite network. Users and items are represented by squares and circles respectively, and solid lines represent the existent links between them. Plot (a) is the original network, and plot (b) is the network after adding the item 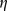 (the yellow circle). The dotted lines are new links connecting 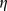 with two existent users.

Recommendation can be considered as a variant of link prediction in bipartite networks [41] and thus the better understanding of network structures can in principle improve the quality of recommendations [42]–[45]. We denote a recommender system by a user-item bipartite network 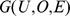, where 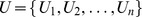 and 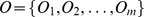 are respectively the sets of users and items, and 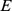 is the set of links connecting users and items. Consequently, we use the adjacent matrix, 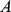, to describe the user-item relations: if user 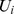 has purchased item 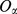, 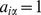, otherwise 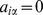 (throughout this paper we use Latin and Greek letters, respectively, for user- and item-related indices). Figure 1(a) illustrates a small bipartite network that consists of eight users (gray squares) and eight items (blue circles). 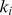, the degree of user 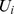, is defined as the number of items linked to 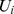. Analogously, the degree of item 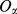, denoted by 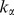, is the number of users connected to 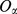. For example, as shown in Figure 1(a), 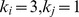 and 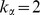. The user degree distribution 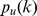, is the probability that the degree of a randomly selected user, is equal to 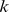, and the survival function, 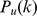, denotes the probability that the degree of a randomly selected user, is no less than 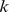. The item degree distribution 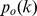 and survival function 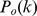 are defined in a similar way. Degree distribution reflects the network heterogeneity [46].

**Figure 2:**
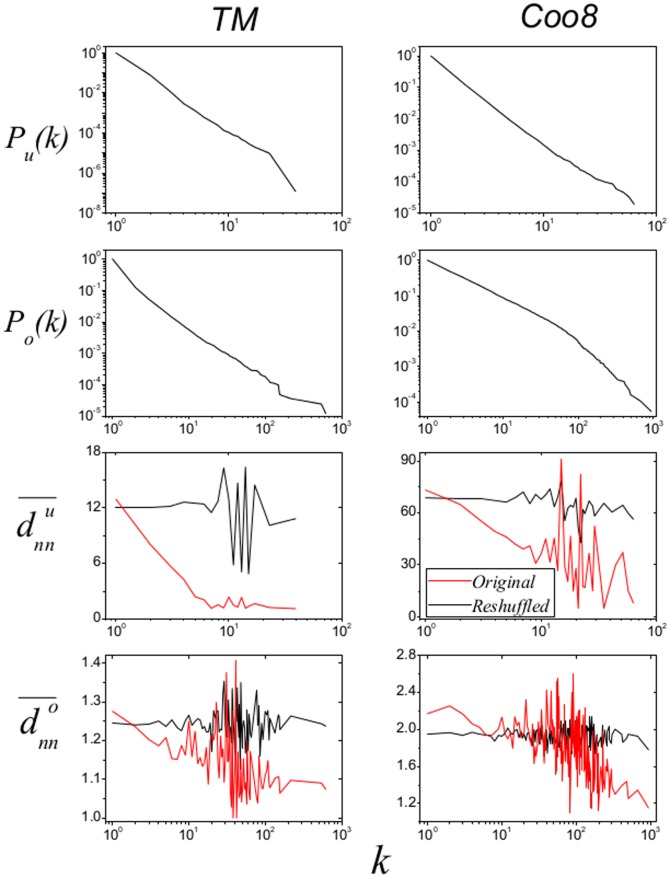
Degree distributions and degree correlations. All degree distributions are power-law-like. 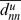 and 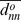 are respectively showed in the 3rd and 4th rows, where red and black lines representing the results from original and reshuffled networks. Results of reshuffled networks are obtained by averaging over five independent realizations.

We consider two real data sets with anonymous users in this paper (datasets are free to download as **Dataset S1**), including (a) *Tmall.com* (TM): an open business-to-consumer (B2C) platform where enrolled businessmen can sell legal items to customers; (b) *Coo8.com* (Coo8): a well established online retailer mainly trading in electrical household appliances and a leading supplier to daily necessities. In order to avoid the isolate nodes in the data sets, each user has bought at least one item, and each item has been purchased at least once. Table 1 shows the basic statistics of the two data sets. Due to the different types of products, these networks have much different average item degrees. As shown in Figure 2, all degree distributions are heavy-tailed and the item degree distributions are generally more heterogenous than the corresponding user degree distributions. These observations complement previous empirical analyses on user-item bipartite networks [47]–[50].

**Table 1:** Basic statistical properties of the two data sets.

Data	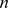	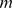	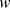	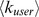	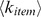	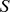
*TM*	103,867	83,342	113,624	1.09	1.36	1.31 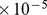
*Coo8*	77,947	18,751	94,457	1.21	5.04	6.46 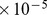

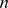
, 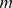, and 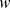 represent the number of users, items and links, 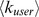 and 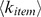 stand for the average degrees of users and items, and 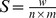 denotes the data sparsity.

The nearest neighbors' degree for user 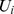, denoted by 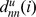, is defined as the average degree over all items connected to 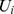 [50]. For example, in Figure 1(a), 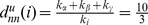. Furthermore, the degree-dependent nearest neighbors' degree, 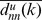 is the average nearest neighbors' degree over all users of degree 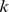, namely 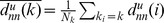, where 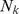 is the number of users with degree 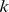. Corresponding concepts for items, 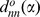 and 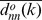 are defined in a similar way and thus omitted here. The degree-dependent nearest neighbors' degree is an appropriate index to characterize the network assortativity [51]. As shown in Figure 2, both the two networks are disassortative.

Recommender systems typically produce a given-length list of unpurchased items for each user based on his/her historical purchases. Of nothing comes nothing, that is to say, it is impossible to predict links for an isolate user or item. So only after having been purchased by some users, an item could have the chance to appear in some other users' recommendation lists. In real e-commerce web sites, to get a new customer is highly costly, and thus under the limited investment, choosing users with considerable coming influence on further recommendations is absolutely critical. Concretely speaking, this problem is described as follow. Given a bipartite network containing 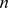 users, 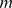 items and 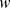 links. A novel item 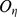 enters this network, and it can at most establish 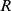 links to users. Given the recommendation algorithm, we need to answer the question that how to choose such 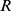 users to maximize the frequency that 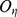 appears in other 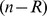 users' recommendation lists. For example, in Figure 1(b), item 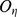 (the yellow circle) comes and needs to link to some existent users. If 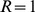, then to choose which user, 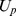 (most active user), 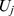 (one of the most inactive users) or another one, can make 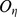 be recommended more times?

We consider four strategies to choose those 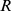 users: (I) Maximum-degree strategy (MaxD). To rank all users in the descending order of degree, and select the top-*R* users, where users with the same degree are ranked randomly. (II) Minimum-degree strategy (MinD). To rank all users in the ascending order of degree, and select the top-*R* users, where users with the same degree are ranked randomly. (III) Preferential attachment strategy (PA). Each user's probability to be selected is proportional to his/her degree. (IV) Random strategy (RAN). The 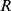 users are selected completely randomly. Actually, all strategies above can be unified by a selecting probability on 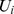 as 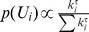, where 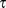 is a tunable parameter. More specifically, the strategies MaxD, MinD, PA and RAN correspond to the cases of 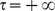, 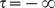, 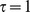 and 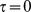, respectively.

Among existent recommendation algorithms, item-based collaborative filtering (ICF) has found the widest applications in real e-commerce platforms for its accuracy, stability, scalability and robustness [5], [6], [38]. Here, we apply cosine similarity for each pair of items, say 
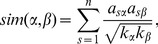
(1)where 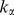 and 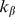 are degrees of items 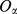 and 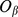, respectively. In fact, the main results are not sensitive to the specific choices of common neighborhood based similarity indices [41], except for some very different indices irrelevant to the common neighbors between two nodes, such as preferential attachment index 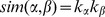. For the target user 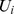, we calculate the accumulative score 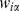 for each item 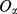 by 
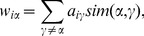
(2)and then rank all the unpurchased items in descending order according to their scores in Eq. (2). The top-*L* items will be recommended to 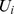, where 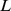 is the length of recommendation list.

To compare the degree-dependent strategies, we employ a metric 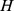 that counts the number of users whose recommendation lists contain the target item (the 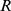 selected users linked to the target item are excluded), say 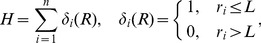
(3)where 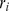 is the position of the target item among all 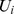 's unpurchased items. Obviously, 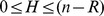, since the target item's degree equals 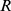, and the larger value of 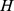 means better performance. The number of recommended items, 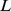, is limited by the user interface, with typical size no larger than 6 (see real recommendation engines of Alibaba Group and Baifendian Inc. as examples).

**Figure 3:**
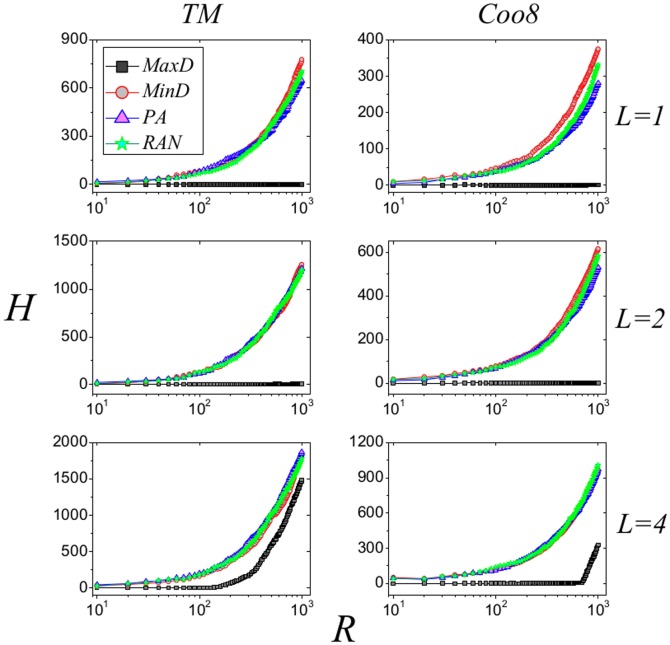
Performance of the four strategies for original TM and Coo8 bipartite networks. The results of MaxD, MinD, PA and RAN are represented by black squares, red circles, blue triangles and green pentagrams, respectively. Data points are obtained by averaging over 100 independent realizations.

In our simulation, we only consider 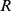 ranging from 1 to 1000 to see the influence of different 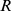 on promoting strategies. It is because too large 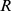 will result in very high cost and indeed 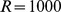 can make the item among the most popular ones. Unexpectedly, as shown in figure 3, MaxD hardly makes new items recommended while MinD usually shows better performance. Consider the general case where the target item 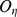 has established a link to user 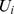, and 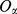 and 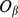 are two of 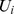 's collected items before 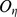. For another user 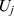 who is not connected with 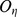. If 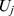 has collected 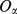 but not 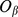, then both 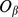 and 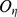 have the chance to be recommended to 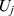. Since in the ICF algorithm, item similarities play the major role, let's compare the similarities 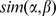 and 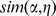. Statistically speaking, if 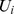 is a very active user selected by the MaxD strategy, 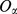 and 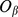 are probably less popular as indicated by the disassortative nature of the networks, therefore 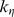 (i.e., 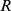) may be much larger than 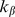 and then 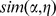 is probably smaller than 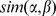, resulting in less probability of 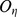 to be recommended to 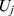. In contrast, if 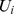 is a very inactive user selected by the MinD strategy, 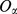 and 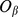 are probably of larger degrees according to the disassortative nature, resulting in smaller 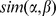 and thus larger probability for 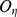 to be recommended to 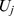. In addition, since 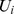 is very unpopular, it is also possible that 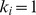 and 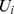 is only connected with 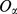. In such case, for all other users connected with 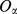, 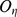 will be the only recommended item related to 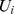.

**Figure 4:**
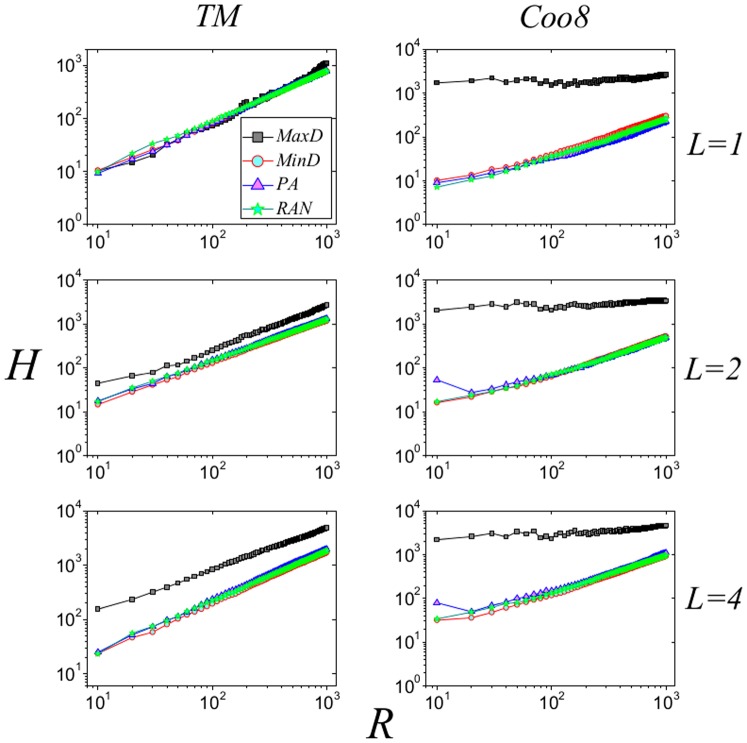
Performance of the four strategies for reshuffled networks. The results of MaxD, MinD, PA and RAN are represented by black squares, red circles, blue triangles and green pentagrams, respectively. Data points are obtained by averaging over 100 independent realizations.

**Figure 5:**
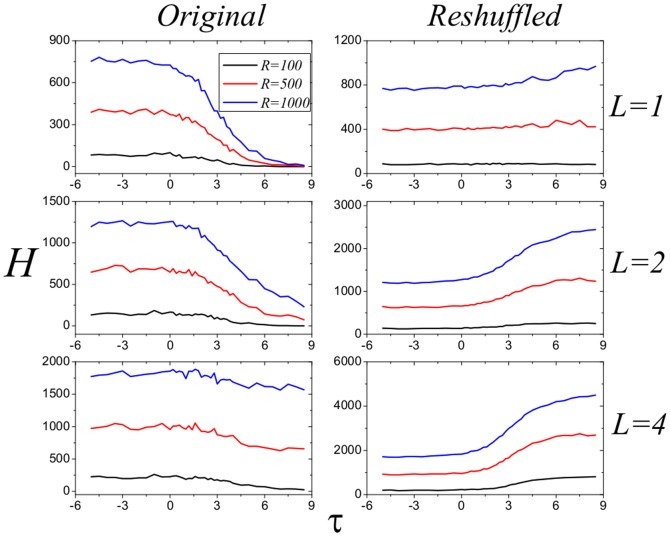
Performance of strategies with different 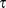 on original and reshuffled TM networks. The black, red and blue lines represent the results for the cases 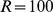, 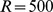 and 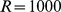, respectively. Data points are obtained by averaging over 100 independent realizations.

**Figure 6:**
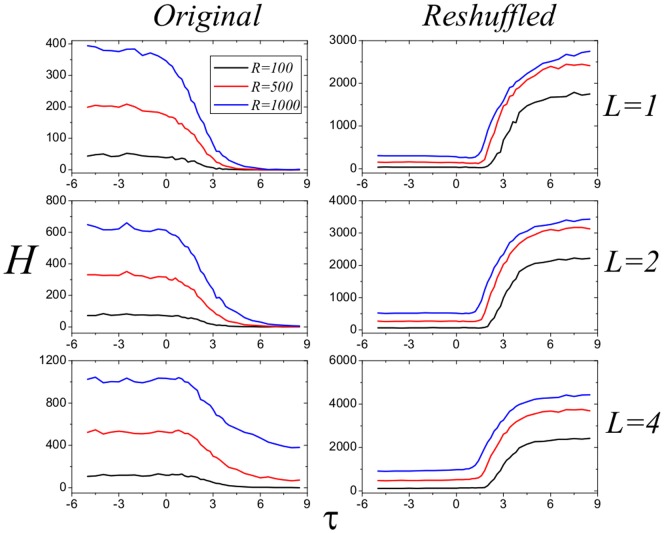
Performance of strategies with different 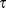 on original and reshuffled Coo8 networks. The black, red and blue lines represent the results for the cases 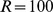, 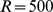 and 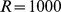, respectively. Data points are obtained by averaging over 100 independent realizations.

In a word, the disassortativity could contribute to the observations in figure 3. To validate this inference, we reshuffle the original networks by link-crossing method to obtain the null networks [52]. Specifically speaking, in each step, two links, say 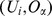 and 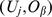, are randomly picked out, and if 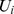 has not collected 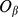 and 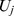 has not collected 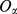, these two links are rewired as 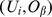 and 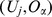. In one realization, we repeat such rewiring for 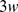 times. After that, the reshuffled network has identical degree sequence as the original network but the disassortative nature is vanished as shown in figure 2. Figure 4 reports the performance of the four strategies in the reshuffled networks, from which we can see that the MaxD strategy performs the best. Comparing the results for original and reshuffled networks, we conclude that the advantage of MinD strategy results from the disassortative nature of real e-commerce user-item bipartite networks. In addition, in figure 5 and figure 6, we test the performance of strategies with different 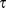. For both TM and Coo8, the negative 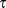 will lead to better performance while in the null networks, positive 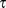 is better.

## Discussion

In this paper, we study a practical problem in e-commerce recommender systems: how to promote cold-start items? Under the item-based collaborative filtering systems, we show that the disassortative nature of real user-item networks leads to a non-trivial observation that to link a cold-start item to inactive users will give it more chance to appear in other users’ recommendation lists. This observation is robust for varying recommendation length 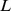 and linking capacity 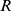. It is also applicative to some variants of item-based collaborative filtering, such as the top-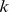 nearest neighbors ICF [5].

Notice that, the reported results are affected by both the topological features and underlying recommendation algorithms. We have tested the user-based collaborative filtering [3], under which the MaxD is usually better than MinD. It is because the high-degree users tend to have high similarities with others, and to connect with those high-degree users can directly benefit items. In spite of this, this work is still relevant since in most real recommender systems, ICF plays a significant role. In addition, the perspectives and methods reported here are useful for real e-commerce applications, with the core merit is that the in-depth understanding of the structure and algorithms of recommender systems can be transferred into applicable knowledge to better market products.

## Supporting Information

Dataset S1 **The TM and Coo8 data sets after anonymization.**
(ZIP)
